# Microstructure and Properties of Tungsten Heavy Alloy Connections Formed during Sintering with the Participation of the Liquid Phase

**DOI:** 10.3390/ma13214965

**Published:** 2020-11-04

**Authors:** Paweł Skoczylas, Zbigniew Gulbinowicz, Olgierd Goroch

**Affiliations:** Department of Mechanics and Weaponry Technology, Faculty of Production Engineering, Warsaw University of Technology, Narbutta 85, 02-524 Warsaw, Poland; pskocz@imik.wip.pw.edu.pl (P.S.); zbigniew.gulbinowicz@pw.edu.pl (Z.G.)

**Keywords:** APFSDS ammunition, segment penetrators, tungsten heavy alloys/weight heavy alloy, sintering with liquid phase, combining in the liquid phase at the sintering temperature

## Abstract

Tungsten heavy alloys (THA) are used in the defense industry for subcaliber bullet cores due to their high density and strength. Typically methods of joining tungsten rod elements include: soldering, friction welding or threaded sleeve splicing. The properties of the joints were tested for three types of material containing 90.8, 96.2 and 98.2 wt.%. tungsten, density from 17.3 to 18.4 g/cm^3^ and strength range 400–1000 MPa. Combination in the liquid phase at the sintering temperature was carried out in a vacuum furnace at a temperature of 1520 °C in a hydrogen atmosphere, and tests used pairs of both identical and dissimilar materials. After that, some of the bars were subjected to additional heat treatment at 1100 °C for 3 h. The tests of the mechanical properties in the static tensile test and the measurement of impact strength showed that the obtained strength of the joints was comparable to that of the parent material. The microstructure analysis showed that the resulting joint area, while maintaining the appropriate roughness of the joined end faces of the bars, is homogeneous without areas of the solidified matrix of the joint line. Research showed that it is possible to bond under sintering conditions with the participation of a solid liquid phase of homonymous and dissimilar THA materials. The strength of joints in dissimilar materials was comparable to a tungsten heavy alloy material with lower strength in the bonded pair while homonymous materials were comparable to the original material. The test results provided a good basis for further research in which the obtained pairs of joints will be subjected to plastic working processes.

## 1. Introduction

Tungsten heavy alloys (THAs) are materials produced mostly by liquid phase sintering. The two-phase microstructure consisting of tungsten grains and a binder causes these materials also to be referred to as tungsten composites [1,2,3,4]. The chemical composition of the matrix is a nickel solution with the addition of elements such as iron, cobalt or copper. Tungsten heavy alloys, due to their characteristic properties such as high density, high strength, plasticity, and impact strength, are used, among others, for the production of radiation shields, balancing elements or sub-caliber bullet cores [5,6,7].

Tungsten heavy alloys were invented by McLennan in 1935 [7]. Attempts to produce THA were carried out to replace lead in the production of radiation shields. In the initial period, the research focused on attempts to obtain products from powdered tungsten without alloying additives, but the production method was not suitable for the production of larger-sized elements. A breakthrough in the research was the use of nickel additive and reduction of the allowable sintering temperature. Nickel as the basic matrix component ensures the wettability and solubility of tungsten in the liquid phase and the reduction of the sintering temperature from 3420 °C (for pure tungsten) to approximately 1520 °C for the W–Ni alloy. The matrix, usually as a solid solution with the main component, nickel, contains dissolved additives such as Fe, Co, Cu, Re, Mo. In addition to them, the solid solution also contains tungsten, the concentration of which in the Ni–W double alloy is 31% (16.5 at.%) [8]. In three and multi-component alloys this depends on the amount of the aforementioned alloying elements. Tungsten enters the matrix as a result of the processes taking place during sintering with the participation of the liquid phase. The size of the tungsten grains after sintering with the participation of the liquid phase is 20–50 µm and depends on the tungsten content in the alloy, the quality and quantity of alloying additives as well as the temperature and time of the sintering process [9,10,11,12]. The produced alloys with the W–5Ni–5Cu composition were characterized by a density close to the theoretical one.

In 1938, Price, Smithells, and Williams [13] used THA in the commercial production of radiation shields for the first time. The shields were made of a material with a Ni–Cu matrix composition and were characterized by low strength and brittleness. Subsequent attempts to replace copper with iron provide an increase in the solubility of tungsten in the matrix, and thus improvement of the mechanical properties of the alloys.

Tungsten heavy alloys with different mechanical properties are used in the production of sub-caliber bullet cores [14,15,16,17,18,19,20,21,22]. The degree of armor penetration by sub-caliber bullets is influenced by many factors. Most are related to the construction of the penetrator, the mechanical properties of the applied materials, the speed and thus the energy at the moment of impact [14].

The cores of sub-caliber bullets made of THA are subjected to heat and plastic treatments in order to improve their mechanical properties, in particular tensile strength and impact strength [23,24,25,26,27,28,29,30,31,32]. High-strength parameters are necessary to prevent the core from damaging during the movement of the projectile inside the barrel. They also affect the piercing capacity of the projectile. The effectiveness of the projectiles increases with their length [15,16]. The greater the ratio of the penetrator’s length to its diameter, the greater the energy that is concentrated on the cross-sectional area during hitting the target. Due to the improvement of armor penetration, the length of the currently manufactured sub-caliber shell cores is up to 30 times the diameter. It is also often necessary to use several different alloys in one element, for example in segment penetrators. An example of a segment penetrator is a core made of two THA materials with different mechanical properties. Therefore, it is necessary to connect these materials, e.g., with a threaded sleeve made of high-strength steel. This design increases the penetration of both homogeneous and multi-layer armor.

Joining parts made of THA may create a detachable or non-detachable connection. The connection method is often determined by technological possibilities, the cost of its implementation, the durability of the link as well as the impact on the work and properties of the entire part.

Threaded connections belong to the group of detachable connections used to connect tungsten alloy rods. Thread milling is required to make a threaded connection. Tungsten is difficult to cut due to its relatively high hardness and strength.

The technologies of permanent connections include: soldering, welding, friction welding and sintering with the participation of the liquid phase [1,33,34,35,36,37].

Soldering can be performed with the use of soft solders, i.e., soldering of easily fusible metals, e.g., lead and tin, at a temperature of up to 450 °C, or hard soldering, i.e., copper, gold or silver soldering at a temperature of 400–2000 °C. Welding is another method of permanent joining of sinters, but it is a technologically difficult operation, because THA is a poorly weldable material. Moreover, the susceptibility to oxidation of tungsten (the O–W (oxygen–tungsten) system) [6,8] at elevated temperature makes the obtained joints porous and not very durable. The welding process should take place in a protective atmosphere to protect the surfaces to be welded against oxidation, and therefore the welding of THA sinters is rarely used.

Frictional rotary welding of THA rods is a possible method. It is also possible to obtain THA connections with other materials. The paper [33,34,35], presents the results of research on friction rotary welding of THA with aluminum (Al_2_Mg_3_). On the basis of the research results, it was found that it is possible to obtain a durable joint by rotary welding. THA can also be used as a welding electrode in friction stir welding.

The paper presents the results of mechanical tests of connections of various THA made at the temperature used in the sintering process of tungsten heavy alloys. Joining in the sintering process will allow a segmented material to be obtained, which later will be heat- and plastic treated as a whole. This process eliminates additional treatments and allows to combine technological materials (separable and inseparable connection). Therefore, the time of the entire technological process was shorten, reducing individual stages and lowering costs.

## 2. Materials and Methods

Three tungsten heavy alloys marked as THA-1, THA-2 and THA-3 were prepared. These materials differed in the content of tungsten and other components, i.e., Ni, Fe, Co. The detailed composition of materials is presented in Table 1.

**Figure 1:**
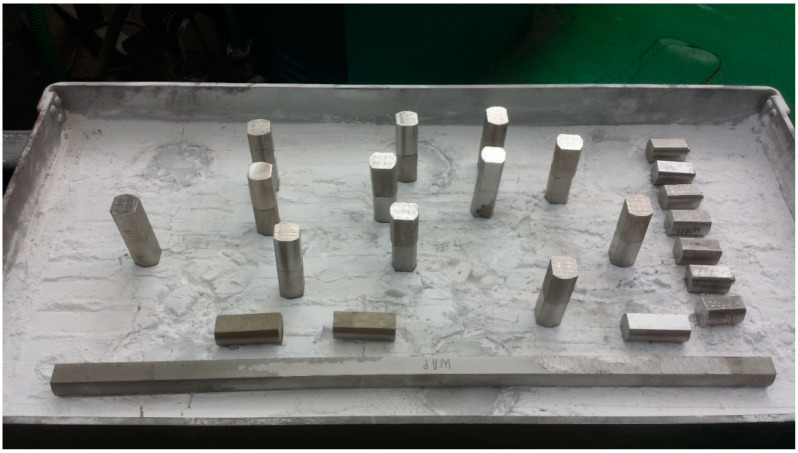
The arrangement of the sections of rods in a molybdenum bath.

Material was prepared by sintering from the liquid phase in the Laboratory of Heavy Alloys Institute of Mechanics and Printing Warsaw University of Technology (Warsaw, Poland). The material compositions selected for the tests allowed for obtaining the appropriate density (from 17.3 to 18.4 g/cm^3^). These are typical materials used in the production of THA rods. This composition results in different densities and mechanical properties of the materials. Materials with a density close to the lower range (17.40 g/cm^3^) show high plasticity and impact strength. The materials from the upper range (18.4 g/cm^3^) show lower strength, no plasticity in deformation and no impact toughness. The process of preparing THA rods [23] included weighing appropriate powder blends containing alloying additives such as nickel, iron, cobalt and tungsten in the amount of 90.8%, 96.2% and 98.2% by weight. Sintering was carried out in a hydrogen atmosphere furnace. The sintering temperature, depending on the tungsten content in the mixture, ranged from 1500 to 1530 °C. The bars obtained in this way, with a diameter of 18 mm and a length of 500 mm, were cut into sections of 30 mm. The front surfaces of the cut sections of the bar were sanded on 120 grating paper and degreased (the results of surface roughness prior to the merger in Table 2). The measurements were carried out on the Form Talysurf Series 2 scanning profilometer. Then, two sections of bars (different configurations of THA) were placed in a vertical position one on top of the other (Figure 1) in a molybdenum bath with Al_2_O_3_ powder. In the case of combining dissimilar samples, the samples with the higher density were placed at the bottom and those with a lower density at the top. With this configuration, when the sample was plasticized at the sintering temperature, the sample with a lower density did not buckle and the upper sample fell from the lower one. The upper and lower samples were pressed by the force of gravity, the mass of one sample was about 130 g.

**Figure 2:**
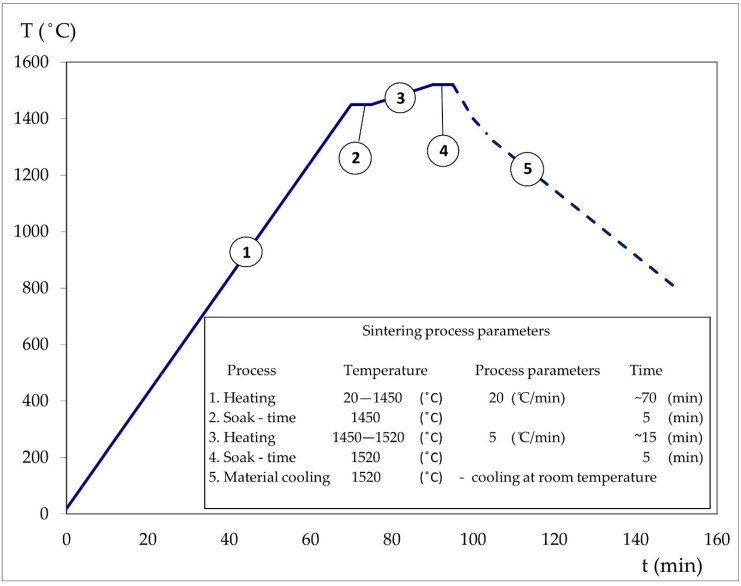
Join-process temperature diagram.

The process of joining (sintering in the liquid phase) the sections of the bars was carried out in the same furnace as the sintering process of the previously obtained THA bars. The process was carried out at 1520 °C for 5 min. in a hydrogen atmosphere. Figure 2 shows the sintering temperature rise ramp. The temperature ramp to 1450 °C was 20 °C/min while between 1450–1520 °C it was 5 °C/min. The sintered samples were freely cooled in a furnace to room temperature. The sections of the rods made of original materials were also kept at the same temperature in order to carry out later tests of mechanical properties. After the process, the joined rod sections and rod sections from original materials were heat treated. The heat treatment–annealing was carried out in a vacuum at 1100 °C for 180 min. Following this annealing process, the rods were removed and flash cooled in water. The final step was water cooling. Metallographic analysis of the joint area, tests of mechanical properties in the static tensile test, and Charpy impact tests were carried out on the samples obtained in the joining process.

The static tensile test was carried out on five-fold round samples with a nominal measuring diameter equal d_0_ = 3 mm in accordance with PN-91/H-04310 (PN-EN 10002-1) on an INSTRON model 1115 testing machine (Instron, Norwood, MA, USA) with a 100 kN head and a traverse speed of 1 mm/min.

Impact tests were performed on samples with dimensions of 10 mm × 10 mm × 55 mm without a notch on a Charpy hammer (VEB Werkstoffprufmaschinen, Leipzig, Germany) with the impact energy of 100 and 300 J. The tests were carried out in accordance with the Stanag 4190 standards (STANAG 4190—Test procedures for measuring behind-armor effects of anti-armor ammunition) and the DIN 50,115 standards [38,39].

The observations of the microstructure were carried out on the Nikon Eclipse MA-200 Microscope (Nikon Corporation, Tokyo, Japan) with the use of 50×, 100×, 200×, 500× and 1000× magnification lenses.

## 3. Results

### 3.1. Analysis of the Microstructure of Joints

The analysis was performed on specimens along the main axis of the rod and perpendicular to the boundary of the joint obtained. Photographs of the microstructure were made in the magnification range of 50–1000× on a Nikon Eclipse Ma-200 microscope.

#### 3.1.1. Connection Type THA-1 with THA-1

**Figure 3:**
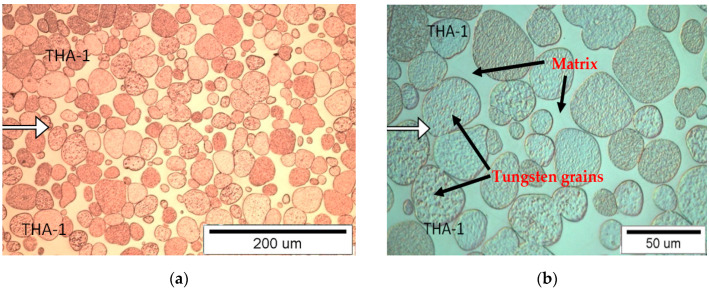
View of the microstructure of the THA-1 with THA-1 at a magnification of 200× (**a**) and 500× (**b**), (the connection line (joint) is shown by the arrow).

The border of the transition from one material to another (Figure 3) is difficult to define in the observed samples. The microstructure in the joint area is homogeneous and comparable with the microstructure in the area distant from the joint boundary. At the border, there are both rounded tungsten grains in the matrix and grains joined by a “neck” from both combined samples, which indicate the processes of dissolving and crystallizing tungsten from the liquid phase and possible diffusion processes of tungsten atoms in the solid phase. The THA-1 material contains less tungsten, so the percentage of matrix in the microstructure is the highest among the three analyzed alloys. This means that material undergoes the greatest plasticity at a combining temperature of 1520 °C. In the case of inaccurate preparation of the joined surfaces and the occurrence of gaps between the joined surfaces, these gaps are not filled by the solidified matrix, but supplemented by the material settling in volume during the process.

#### 3.1.2. Connection Type THA-2 with THA-2

**Figure 4:**
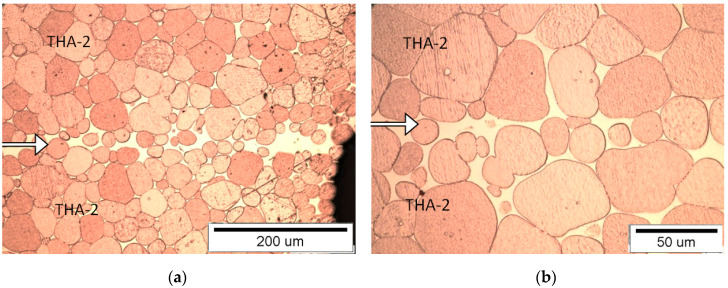
View of the microstructure of the THA-2 with THA-2 at a magnification of 200× (**a**) and 500× (**b**), (the connection line (joint) is shown by the arrow).

Due to the higher content of tungsten than in the THA-1 sample, the material is less plastic, so there are visible areas of a solidified matrix filling the gaps between the joined surfaces at the side edges of the sample (joints) (Figure 4). If joined surfaces are well matched, there is little or no precipitation of the binding phase in the joint. The connection boundary is homogeneous. New crystallized tungsten grains and primary tungsten grains from both materials connected to the neck in the joint area are visible in the border area.

#### 3.1.3. Connection Type THA-3 with THA-3

**Figure 5:**
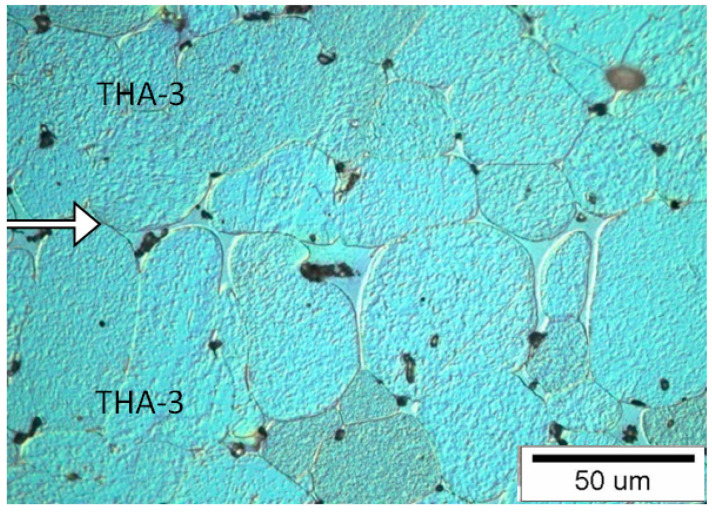
View of the microstructure of the THA-3 with THA-3 at a magnification 500× (the connection line (joint) is shown by the arrow).

This material contains the greatest amount of tungsten, which leads to a reduction of the matrix. The material is difficult to plasticize at the combining in the liquid phase at the sintering temperature. Small gaps between the surfaces are connected by the solidified matrix. In the case of a good match of the joined surfaces, the joint line is hardly noticeable, without the areas of the solidified matrix. Both materials (Figure 5) are connected mainly by tungsten grains flattened at the joint with each other. The flattened tungsten grains at the joint with each other are probably separated by a thin matrix layer, the chemical composition of which may be different from that of the matrix in the parent material. There are single tungsten grains jointed by a neck in the area of the matrix.

#### 3.1.4. Connection Type THA-1 with THA-3

**Figure 6:**
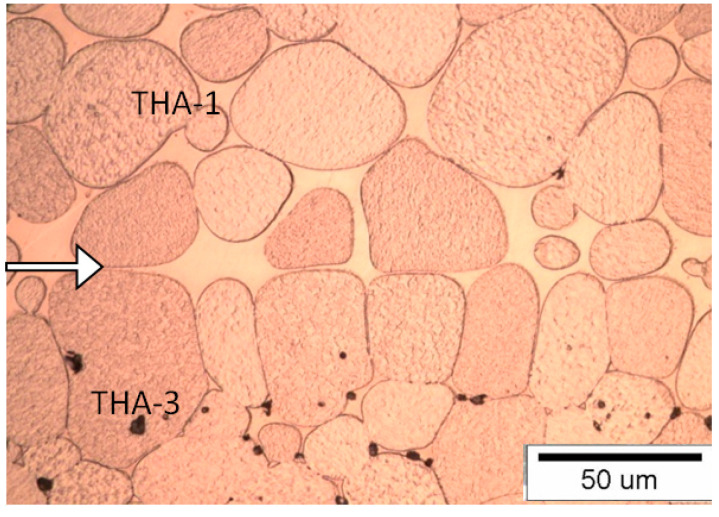
View of the microstructure of the THA-1 with THA-3 (THA-1 on the top) at a magnification 500× (the connection line (joint) is shown by the arrow).

The first combination among mixed (dissimilar) joints is where the joint line is clearly visible due to the microstructure and size of the tungsten grains in both materials. The tungsten grains in the THA-3 material are flattened at the point of contact with the solidified matrix of the THA-1 material (Figure 6). On the THA-1 side surfaces of the sample, areas with increased concentration of the matrix are visible. The joint line includes both contacting grains from both materials and tungsten grain–matrix joints. Crystallized rounded tungsten grains in the matrix of the THA-1 material are also visible. Due to the different amount of tungsten in both alloys, the THA-1 alloy has a visibly greater amount of matrix, while the THA-3 alloy contains the largest amount of tungsten (grains). As a result, the amount of matrix is minimal, and the tungsten grains almost touch each other, and are not separated by the matrix. This makes it easier to recognize the boundary between the materials. The THA-1 material is at the top and THA-3 is at the bottom of the picture.

#### 3.1.5. Connection Type THA-1 with THA-2

**Figure 7:**
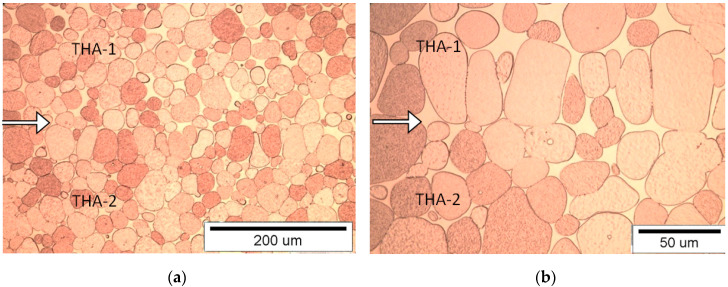
View of the microstructure of the THA-1 with THA-2 (with THA-1 on the top) at a magnification of 200× (**a**) and 500× (**b**), (the connection line (joint) is shown by the arrow).

Due to the difference in tungsten grain size and share in both matrix materials, the boundary of the connection is clear and it is particularly easy to observe the pictures at a magnification of 50×. There are crystallized tungsten grains and grains connected by a neck in the area of the joint (Figure 7). In the area of the joint in the THA-1 material, there are flattened, solidified areas of the matrix with evenly spaced joint lines. THA-1 material is at the top and THA-2 is at the bottom of the picture.

#### 3.1.6. Connection Type THA-2 with THA-3

**Figure 8:**
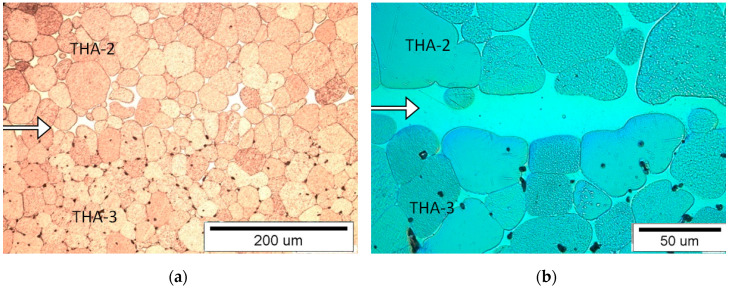
View of the microstructure of the THA-2 with THA-3 (with THA-2 on the top) at a magnification of 200× (**a**) and 500× (**b**), (the connection line (joint) is shown by the arrow).

Connection line between the materials is clear (Figure 8). Areas with both matrix separations and without visible matrix separations can be observed at the border. If there are gaps between joined surfaces, they are filled by a matrix out of the THA-2 material. The tungsten grains in both materials are flattened at the interface with the matrix. THA-2 is at the top and THA-3 is at the bottom.

### 3.2. Strength Tests

#### 3.2.1. Static Tensile Test Testing

The aim of the research was to determine the basic mechanical properties, i.e., tensile strength R_m_, yield stress R_p0.2_, elongation A_5_. The research was carried out on 3 samples from each state (Table 3).

**Figure 9:**
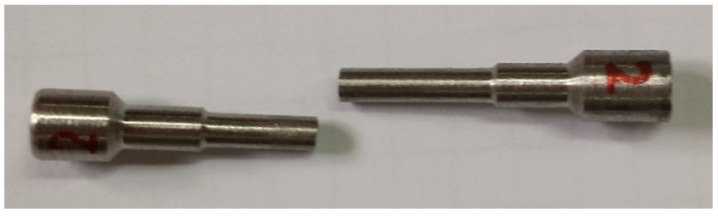
Sample THA-2 with THA-3 fractured in the joint line or in its immediate vicinity.

**Figure 10:**
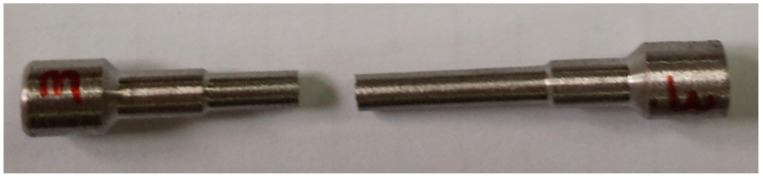
Sample THA-1 with THA-3 fractured in the zone distant from the joint line material THA-3.

One of the main problems of the tests was to determine the strength of the joint and whether the sample broke in the joint (Figure 9 and Figure 10). The samples were prepared in a such way that the connection area was in the middle of the sample measuring section.

The tensile strength of the samples from THA-1 material was 990 MPa, 944 MPa for THA-2 and 680 MPa for THA-3. The strength of the combined homonymous materials is comparable to the strength of samples from original materials. The THA-3 material (THA-3 with THA-3 where the strength is about 25 MPa lower than the strength of the base metal), due to its microstructure, has the largest dispersion of results in strength tests. For this material, a greater difference of the average value between the strength of the base material and the joint by nearly 25 MPa was noticed. This difference is within the error range, and therefore the materials are comparable.

On the basis of optical inspection, it was found that there were only three breaks in the join or in its immediate vicinity among 27 samples. These include two samples of THA-3 with THA-3, and one sample THA-2 with THA-3. The probable cause of the breakage of sample THA-2 with THA-3 was the gap that was not filled with the matrix during the process.

For samples of dissimilar joints, the resulting strength is comparable to the strength of the material of lower strength used in a bonded pair, as evidenced by scrap present in these materials.

#### 3.2.2. Impact Test Results

Impact tests were carried out on a Charpy pendulum hammer on samples with dimensions of 10 mm × 10 mm × 55 mm without a notch. To determine the strength of the joint and the areas around the joint, the samples were hit with a beater at two points (lines): in the joint or 5 mm from the joint. To this end, the samples were ground and etched to determine the location of the joint. The apparent boundary between the two materials were determined by a red or blue border around the perimeter of the section of the sample. The results of the impact tests are presented in Table 4. Initially, the tests were carried out using a hammer with an energy of 150 J. Due to the fact that some of the samples did not break, a second hammer with an energy of 300 J was used.

The highest impact toughness was achieved for THA-1 homonymous connections. In the first test, a hammer with an energy of 150 J was used, hitting a line 5 mm from the joint at which the sample did not break (Charpy Impact Energy > 150 J/cm^2^). In the second test, a 300 J hammer was used, hitting the joint, and the sample also did not break (Charpy Impact Energy > 300 J/cm^2^). In the third test, hitting the impactor with an energy of 300 J, the sample broke next to the place of impact, which means that the strength of the joint is higher than that of the area near the joint. A separation fracture was observed on the sample, the sample was bent, the crack occurred beyond the yield point, i.e., with significant permanent deformations. Significantly lower results were obtained for material of the THA-2 type. Examination of the original material showed the impact strength of the order of 33–36 J/cm^2^. In case the THA-2 joint, the result was 15 J/cm^2^ hitting the joint and 32 J/cm^2^ hitting 5 mm from the joint. The reason for the lower impact resistance against the joint is probably due to the inhomogeneity of the joint (gaps) obtained, the type of material and its hardness. In the case of testing dissimilar joints, THA-1 with THA-2, the result of impact on the joint was 56 J/cm^2^. The impact strength of dissimilar THA-1 with THA-2 joints depends on the place of impact. The result over 150 J/cm^2^ were obtained in case hitting 5 mm from the joint THA-1 and 76 J/cm^2^ for hitting the THA-2. More than twice the value of impact strength (between THA-1 and THA-2) is probably related to the heterogeneity of the material. A separating fracture was observed on the sample, the crack occurred beyond the yield point, i.e., with significant permanent deformations.

The THA-3 material is a brittle fracture material that does not show any plasticization before breaking in the tensile test. Due to its intended use, this material requires only a certain tensile strength, not impact strength.

The results of the impact test depend mainly on the chemical and phase composition of the metallic material, the type of hammer and sample, and the temperature of the test.

The low impact toughness of THA-1 with THA-2 and THA-2 with THA-2 is probably associated with a large extent of phase boundaries in contact with tungsten grains from both materials. The test results presented in the tables indicate that the samples usually broke at the point of impact.

The dimensions and shape of the impact samples have a large influence on the results of the impact test of the tested material. The results of the impact test depend mainly on the chemical and phase composition of the metallic material, the type of hammer and sample, and the temperature of the test. All the factors that contribute to the increase in the hardening of the structure also contribute to the reduction of toughness. The presence of brittle phases in the structure, separated from the grains, has a particularly negative impact on the result of the impact tests. The effect of this is the occurrence of an intercrystalline fracture. Solid solutions have greater impact strength than mixtures and supersaturated or strain-hardened solutions.

## 4. Discussion

The core of a sub-caliber projectile consists of several elements that can be joined, inter alia, by soldering, welding or an intermediate sleeve. A characteristic feature of this method of making connections is the use of THA rod sections with mechanical properties obtained by a combination of heat and plastic treatments. The aim of the research conducted was to determine the mechanical and structural properties of similar and dissimilar connections of THA materials. The combination of both materials was achieved using the temperature employed in sintering tungsten heavy alloys. The tests conducted showed that due to the maintenance of flatness, parallelism of the joined surfaces and similar roughness, the joints obtained are structurally homogeneous, without the presence of gaps or the presence of a solidified matrix along the border line of the joint. The tests of mechanical properties in the static tensile test and in the impact tests showed that in the case of homonymous connections, the strength of the joint is comparable to that of the original material. However, in the case of dissimilar joints, the strength of the joint is comparable to the strength of the THA material with lower parameters used in the joined pair. In the case of properly prepared joint surfaces, the connection line for homonymous materials is difficult to define. For dissimilar materials, the joint line can be determined mainly by differences in the grain size and matrix amount of the parent materials. When used at the temperature of 1530 °C, the material becomes plasticized and a liquid phase is formed. The processes of dissolution and crystallization of tungsten grains taking place in the liquid phase can lead to the formation of a homogeneous microstructure consisting of tungsten grains and a matrix without the additional presence of a solidified matrix layer. It is also possible that the binding phase layer is so thin that it has not been observed under microscopic observations.

Positive results are the basis for further research and analysis of joints obtained at the sintering temperature and then subjected to heat treatment and plastic working. The conducted tests are innovative attempts to combine various types of tungsten heavy alloys used in sub-caliber ammunition. The use of THA cores with gradually variable tensile strength, impact strength, plasticity and density will allow the external and final ballistics properties of sub-caliber bullets to be improved.

## 5. Conclusions

Based on the research, it can be concluded that:

- It is possible to bond the same and dissimilar materials under sintering conditions with the participation of the liquid phase.
- In homonymous materials, the microstructure in the joint area is homogeneous and does not differ in morphological features from the area further from the joint area, which makes location difficult to determine correctly.
- The strength of the combination of homonymous materials is comparable to the original material. The strength of joints in dissimilar materials is comparable to that of a tungsten heavy alloy material with lower strength used in the joined pair.
- In the case of materials with properly prepared front surfaces (no gaps), a joint is obtained without a layer of solidified matrix.
- Determining the location of the connection line in dissimilar materials is easier to determine due to the size of tungsten grains in both materials and the amount of matrix.
- The results obtained constitute the basis for the continuation of further work, including subjecting the obtained joints to plastic cold forging.
- The homogeneity of the microstructure and the lack of a visible boundary between the materials are the result of dissolution and crystallization processes taking place in the liquid phase.

## Figures and Tables

**Table 1:** Composition of materials used for research on tungsten heavy alloys (THAs).

	THA-1	THA-2	THA-3
Density (g/cm^3^)	17.40	18.44	18.53
W (%)	90.8	96.2	98.2
Ni (%)	6.2	2.8	1.4
Fe (%)	1.2	0.8	0.4
Co (%)	1.8	0.2	0

**Table 2:** The results of surface roughness prior to the merger.

	THA-1	THA-2	THA-3
Ra (µm)	0.30 0.28 0.28	0.32 0.31 0.32	0.34 0.34 0.34
Rz (µm)	2.42 2.20 2.36	2.51 2.49 2.47	2.95 2.67 2.95
* Wa (µm)	0.28 0.27 0.27	0.32 0.30 0.32	0.33 0.34 0.57

* Wa—surface waviness measured at 10 mm.

**Table 3:** The results of the strength properties research.

A Sample	R_m_ (MPa)	R_p0.2_ (MPa)
THA-1/THA-1	980 ± 9	645 ± 7
THA-2/THA-2	945 ± 8	681 ± 6
THA-3/THA-3	655 ± 20	580 ± 8
THA-1/THA-2	942 ± 10	650 ± 8
THA-1/THA-3	675 ± 13	569 ± 11
THA-2/THA-3	670 ± 15	554 ± 12
THA-1	990 ± 6	647 ± 9
THA-2	944 ± 6	688 ± 9
THA-3	680 ± 15	585 ± 10

**Table 4:** Impact test results.

Sample No.	Types of Combined Materials	Impact Strength (J/cm^2^)	Place of Impact	the Place of the Crack
1	THA-1 with THA-1	>150	5 mm from the joint	It was not broken
2	THA-1 with THA-1	>300	joint	It was not broken
3	THA-1 with THA-1	190	joint	Next to the point of impact
4	THA-2 *	33	-	At the point of impact
5	THA-2 *	36	-	At the point of impact
6	THA-1 with THA-2	56	connector	At the point of impact
7	THA-1 with THA-2	76	5 mm from the joint on the THA-2 side	At the point of impact
8	THA-1 with THA-2	150	5 mm from the joint on the THA-1 side	At the point of impact
9	THA-2 with THA-2	15	joint	At the point of impact
10	THA-2 with THA-2	32	5 mm from the joint	At the point of impact

* original material.
